# Who Speaks for Science?

**DOI:** 10.1007/s11191-021-00257-4

**Published:** 2021-10-16

**Authors:** Douglas Allchin

**Affiliations:** Minnesota Center for the Philosophy of Science, University of Minnesota, Minneapolis, MN USA

**Keywords:** Science media literacy, Credibility, Expertise, Science con artist, Deceptive practices

## Abstract

Ironically, flat-Earthers, anti-vaxxers, and climate change naysayers trust in science. Unfortunately, they trust the *wrong* science. That conundrum lies at the heart of scientific literacy in an age of well-funded commercial and ideological interests and overwhelming digital information. The core question for the citizen-consumer is not philosophically “why trust science?” (Oreskes 2019) but sociologically “who speaks for science?” Teachers can help students learn how to navigate the treacherous territory of inevitably mediated communication and the vulnerabilities of epistemic dependence. Students need to understand the role of science communication practices (media literacy) and the roles of credibility, expertise and honesty and the deceptive strategies used by imitators of science to seem like credible voices for science.

## Introduction

The bane of science used to be pseudoscience (e.g., Feder, 1999; Gardner, 1957; Park, 2000; Shermer, 2002). Now it’s conspiracy theories, fake news, alternative facts, manufactured uncertainty, misinformation, *dis*information, and the grotesque bending of science by industry, politicians, and ideologues (Jackson & Jamieson 2007; Levitan, 2017; Markowitz & Rosner, 2002; McGarity & Wagner, 2008; Michaels, 2008, 2020; Oreskes & Conway, 2010; Otto, 2016; van Prooijen, 2018). Large numbers of people deny anthropogenic climate change, reject the safety of vaccines, and dismiss the scope and severity of the coronavirus pandemic. Flat-Earthers are on the rise. There seems to be a widespread lack of trust in science, hence the focus of this thematic issue. What is the remedy? What is the role for science educators?

A widespread view among philosophers and educators is that we need to redouble our efforts at teaching critical thinking. We need to deepen the philosophical appreciation of science as a “special way of knowing” and (as in battling pseudoscience) help students demarcate science from other ways of knowing (e.g., Daempfle 2013; Gardner, 1957; Lange, 2019; Matthews, 2019; McIntyre, 2019; Pigliucci, 2010; Pigliucci & Boudry 2016; Shermer, 2017). We apparently need to bolster skepticism and promote epistemic vigilance. We need to develop skills in evaluating evidence and in avoiding the common fallacies of reasoning. Only in this way, it seems, can students become autonomous, independent thinkers, able to debunk and reject the unfounded claims of science deniers (Agin, 2006; Bergstrom & West, 2020; Cromer, 1993; Helfand, 2016; McIntyre, 2019; Popper, 1962; Wolpert, 1992; Zimring, 2019).

Here, I argue otherwise. The central problem, I contend, is not justifying or bolstering trust in science, as elegantly addressed by Naomi Oreskes in *Why Trust Science?* (2019). Rather, analysis of the science contrarians indicates that they do, indeed, trust science (first section below). Unfortunately, they trust the *wrong* science. Or, perhaps more appropriately, their sources of scientific information do not report scientific consensus. They trust the wrong *voice* for science. To address the question of scientific misinformation, then, educators must shift their focus from *what* to trust to *who* to trust. Educators must replace the abstract philosophical orientation of articulating general scientific practices and “why trust science?” with a concrete psychological and sociological one of mediated scientific information in society—namely, “who speaks for science?”.

The shift from what to who introduces several challenges for educators. This involves, at least, shifting from science-centered communication to consumer-centered scientific literacy (Sect. 2); from the workings of experts within science to the epistemic problems of non-experts in public discourse (Sect. 3); from experimental reasoning to the social concepts of credibility, expertise, honesty, and transparency (Sect. 4); and from scientific practices to science communication practices (Sect. 5). The goals of science education thus need to broaden significantly from scientific literacy to include science media literacy and to address the reliability of scientific claims wherever they appear, from test tubes to YouTube, from lab book to Facebook (Höttecke & Allchin, 2020).

## Interpreting “Anti-science” Beliefs: Irrationality or Deception?

Why do people believe claims that scientists regard as utter nonsense? Why do they not accept arguments based on extensive evidence? Why do they fail to respect the consensus of scientific experts? How are we to understand “anti-science” beliefs—and based on what evidence?

Consider, for example, the recent case of the coronavirus pandemic that emerged in early 2020. Research resources were rapidly remobilized around the globe, aiming to understand the new disease—its transmission patterns, the virus’s genome, and its molecular components—with findings in remarkably short time. At the same time, spurious theories about the origin and cause of the virus circulated widely on the Internet and on social media. One prominent claim attributed the health effects to radiation from the new 5G telecommunications network. No matter that this view was not endorsed by professional scientists. 5G cell towers were vandalized and burned across Europe (Hamilton, 2020; Sorkin, 2020; van Prooijen, 2020). Given the urgency of solving a shared public health crisis, what guided these individuals to adopt such an ill-informed claim? Meanwhile, national leaders in the USA and Brazil touted an untested remedy (a “game changer,” one said), even while veteran infectious disease specialists cautioned that there was only anecdotal evidence, at best. (A quickly assembled clinical trial in Manaus, Brazil, was soon halted when fatal side effects emerged.) The COVID-19 episode illustrates that a huge gulf exists between the consensus view of scientists and what a meaningful proportion of the populace *accepts* as justified science. Is this the result, as many contend, of the Internet and social media technologies (e.g., Avaaz, 2020; O’Connor & Weatherall, 2019)?

While such cases are alarming, they are not unprecedented. Evidence from history can be informative, here. One could equally have cited the case of New Madrid, Missouri, a town that closed schools and prepared for an earthquake on December 3, 1990, based on a spurious prediction by someone with absolutely no relevant expertise and no concrete evidence (Spence et al., 1993). Or over two million voters in California who approved a 1986 ballot referendum for mandatory HIV testing and quarantining, based on the mistaken belief that HIV could be transmitted by casual contact (Toumey, 1996, pp. 81–95). Or the anti-fluoridationists of the 1950s and 1960s who paraded studies about the dangers of fluorosis (Martin, 1991; Toumey, 1996, pp. 63–80). Ironically, all these “scientific” debates thrived largely outside scientific journals. Science and *what counts as* science in the public realm have diverged dramatically even in the past, without digital media (Allchin, 2012c).

The proposed reasons for the current crisis are many. Many advocates of science conspicuously denounce the popular views as *anti-science* and place responsibility for them squarely on the shoulders of the individual consumers of science. Beliefs that contradict science, they contend, arise from willful ignorance, gullibility, wishful thinking, self-delusion, pure folly, and the like (e.g., Agin, 2006; Forgas & Baumeister, 2019; Gratzer, 2000; McIntyre, 2019; Shermer, 2002). *If only* non-scientists made an effort to be more rational! Others will target poor science communication (e.g., Petersen et al., 2019). *If only* scientists could learn to speak to the public better and explain their conclusions—and give it a human face—somehow sentiments and deference towards science would be better! Others target deficits in science education. *If only* students learned the “nature of science” (NOS) and how science worked, they would *understand* science and thus believe only valid, empirically demonstrated conclusions! *If only* students were steeped in “scientific practices,” or in argument, logic, and the rules of evidence, they would become independent agents, equipped to address all the controversial claims of science and society themselves and sort bonafide claims from junk (e.g., Erduran & Jiménez-Aleixandre, 2008; NGSS Lead States, 2013; Osborne, 2010). Yet others lay the blame on poor “science marketing” (e.g., Lindee, 2019). *If only* we did a better job “selling” the benefits of technology and the triumphs of scientific discovery to the public, the skepticism and anti-science attitudes would soon fade away, to be replaced by proper respect! *If only!*

If only? Alas, all these popular interpretations and widely endorsed solutions lack evidence. They are speculations, with little research to show that remedying any of these alleged lapses will restore functional scientific literacy. Rather, they all appeal to idealized norms, which are merely assumed to be the critical factors in how citizen-consumers interpret scientific claims in actual cultural contexts.

**Fig. 1:**
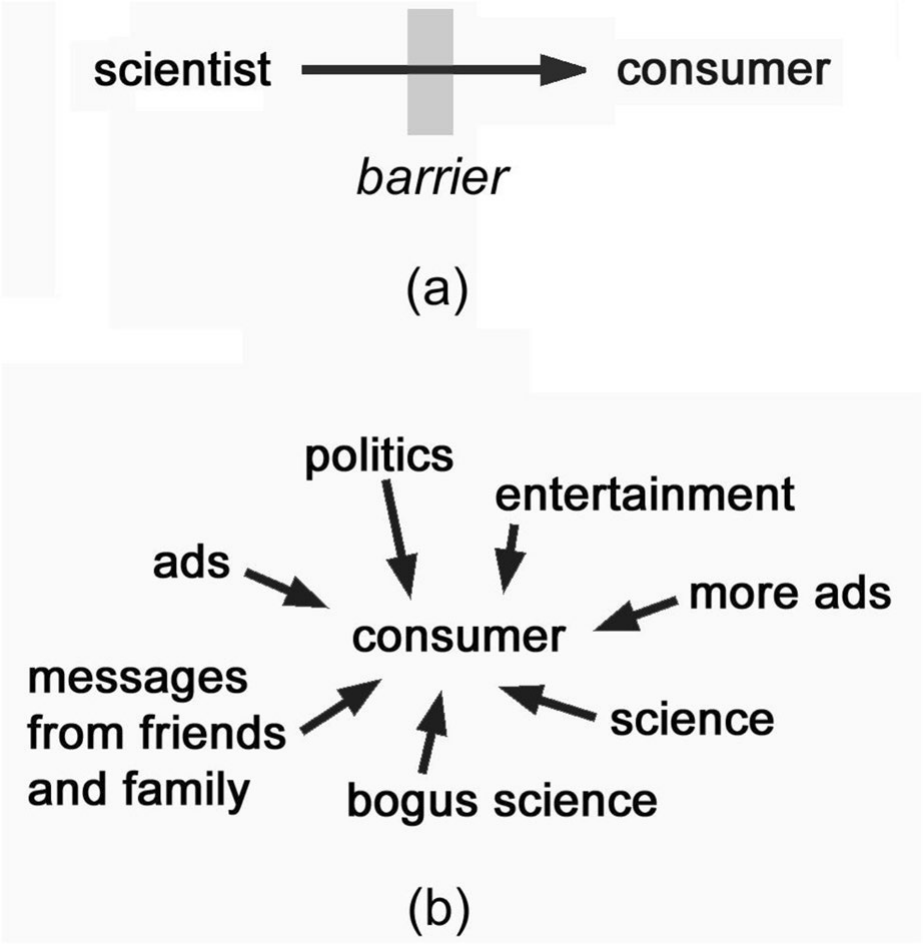
Two models of science communication. **a** Barrier model, **b** consumer-centered model

A realistic model of public understanding of science is needed. For the most part, science communication is conceptualized as linear: a single channel of information flow, linking scientist to citizen-consumer. The goal of education and science news media is to get the channel to function well. All the solutions catalogued above imagine barriers to this flow, which, “if only” removed, would presumably allow information to flow as it “should.” Accordingly, one may call this the *barrier model* (Fig. 1).

Here, educators may draw on familiar learning theory to interpret the challenge. Teachers are well aware of the importance of student-centered learning. However, all the efforts above aimed at securing trust in science notably adopt the perspective of a science advocate. In constructivist pedagogical terms, they are speaker-centered (teacher-centered). We need, instead, to reorient understanding of science communication to a *consumer-centered model* (Fig. 1). We must interpret the citizen in the midst of a barrage of information, only some of which is authentic science. How does the individual select information? How does one discern “good” information from “bad” information?

In the science classroom, an instructor hoping to reshape thinking must first understand (sympathetically) just how a student thinks or misconceives a target concept. They must then create an instructional trajectory or learning sequence that will help them notice the flaws in any particular conceptualization and guide them towards a deeper and more informed perspective. From a constructivist perspective, one can appreciate how utterly counterproductive it is to treat a creationist student, say, as self-deluded or willfully ignorant (Allchin, 2013). That is not an effective strategy for achieving conceptual change. Just so for science communication in a cultural context.

Getting inside the perspective of those who reject consensus science is decidedly discomforting. One must risk acknowledging that “irrational” views may *seem* reasonable or even compelling. But if one has the courage to listen sympathetically, one will soon discover that the problem of the so-called science denial is plainly not denial of science. Indeed—paradoxically perhaps—the “deniers” often vigorously appeal to science. They *do* trust science. Alas, they trust the *wrong* science. They nonetheless feel that the science is “on their side.”

Several concrete cases may illustrate. For example, as evidence for the case of 5G causing COVID-19, one might be advised to compare maps that show the outbreaks of COVID-19 and the locations of 5G towers. There was a striking and unmistakable correlation. One can easily find testimony elsewhere of the harm of cell phone radiation. For instance, an elementary school in California was shut down in March, 2019, after several students were diagnosed with cancer (Carlson, 2019). That echoes an Italian Supreme Court judgment that a plaintiff’s brain cancer was caused by using his work cell phone (Owens, 2012). In 2011, the International Agency for Research on Cancer classified radiofrequency radiation as a Group 2B carcinogen. Thus, the anti-5G protestors’ placards proclaim, “Have you heard of SCIENCE?” and “We Believe in SCIENCE: Wireless Radiation Is Harmful to Your Health.” All this evidence is scientifically incomplete and misleading, of course. But it conspicuously indicates that there is no radical dismissal of the authority of science. Rather, it shows how the dissenters seek, ironically, to leverage that authority in favor of their own claims.

What about flat-Earthers? They, too, appeal to science—and to philosophy of science. Yes—as amazing as it may seem to the “outsider”—the Flat Earth Society advocates empiricism and epistemological reflection. Consult their online wiki (wiki.tfes.org). They assert that:

there is a difference between believing and knowing. If you don’t know something, and cannot understand it by first principles, then you shouldn’t believe it. We must, at the very least, know exactly how conclusions were made about the world, and the strengths and weaknesses behind those deductions. Our society emphasizes the demonstration and explanation of knowledge.

They invite you (the naive initiate and skeptic) to find evidence by “relying on one’s own senses to discern the true nature of the world around us. … This is using what’s called an empirical approach, or an approach that relies on information from your senses.” It all resembles rhetoric you might find in an introductory philosophy of science textbook. On their webpage on “Experimental Evidence,” you will find descriptions of what must be regarded as several “classic” experiments: the Bedford Canal Experiments (on water convexity), the Bishop Experiment, and several dozen others, which (they proudly remind you) are reproducible. So, one might take care, perhaps, before prejudicially discounting a flat-Earther as a science *denier* or failing to have *trust* in science.

The same is true in other purportedly “anti-science” cases. Opponents of the MMR vaccine highlight that diagnoses of autism are strongly correlated with the administration of vaccines only months earlier. They will also note the obvious conflict of interest in the for-profit pharmaceutical industry, whose assurances about safety can surely be discounted (e.g., ageofautism.com). Likewise, climate change naysayers have assembled extensive websites that compile and proudly present all the scientific evidence against the (so-called) consensus (for example, WattsUpWithThat.com, climateaudit.com, or CO2science.org). Creationists, for their part, are only too happy to take aim at the least *scientific* flaw in evolutionary theory (e.g., Jonathan Weiner’s *Icons of Evolution*). Intelligent design advocates (such as Michael Behe or William Demski), despite their caustic criticisms of evolutionary science as “secular humanism,” desire nothing more than to establish their own credibility through *scientific* legitimacy. Historically, both sides of the fluoridation debate have appealed to science as triumphing for their side (Martin, 1991; Toumey, 1996). Trust in science is not the issue. Their core arguments, ironically, seem to embody trust in science.

By now, general public trust in science has long been well established (e.g., Smith & Sou, 2013). It is integral to national and international policy, on everything from the safety of food, drugs, and consumer products to defense systems, health care, and managing potential environmental risks. Nowadays, the battle lines have shifted to the struggles about who gets to claim that authority (Gieryn, 1999; Jasonoff, 1990). The critical social question is no longer “why trust science?” but “who speaks for science?”.

Accordingly, many parties who want cultural authority—politically, ideologically, and commercially—are prepared to exploit the widespread trust in science. Special interests target non-experts who *trust* science but at the same time are not equipped to *distinguish* good science from bad science. As Oreskes (2019) notes, there are many purveyors of *facsimile* science or *sham* science (pp. 240–241). Thus, belief in bad science does not indicate a failure to trust science. Rather, it is a failure to differentiate authentic expert claims from junk crafted to look like science: a very different problem indeed.

The core problem thus seems to be deceit and deception (e.g., Markowitz & Rosner, 2002; Rampton & Stauber, 2001). As aptly described by Christopher Toumey, the imitators “conjure” science “from cheap symbols and ersatz images” (1996, p. 6). The purveyors of scientific misinformation use shards of evidence. Selective data gives an *impression* of empirical confirmation. They use numbers and graphs, even if with bias. Their arguments use statistics. They appeal to nature-of-science concepts like tentativeness and falsification. All in order to *look* like science, and thus capitalize on an underlying trust in science, writ large. Notably, these “scientific” claims rarely enter the professional discourse. They dodge the scrutiny and criticism of recognized experts. That is, the superficial appearance of science in *public* discourse seems to substitute for engagement in real science. Again, there is no general lack of trust in science. The chief problem nowadays is mistaking imitators of science for the real thing. Deception not science denial or irrationality.

## Assessing Evidence vs. Assessing Credibility

How is the individual citizen or consumer to sort fact from faux fact? Ironically, the non-expert seeks an expert’s informed perspective precisely because they do not have the expertise to determine the facts themselves. They need an expert. This asymmetry in expertise poses a puzzling conundrum: how do you cross-check someone’s expertise without knowing the facts first? And it underscores a further challenge: the acquisition of almost all our knowledge is *mediated*.

At first, one might easily imagine that the citizen-consumer, as a non-expert, should learn the same epistemic practices and principles as scientists: the very ones that ultimately justify the claims—namely, via evidence and logical argument. Over the years, philosophers have articulated the many possible sources of error and the corresponding methodological remedies—calibrations, experimental controls, statistical analysis of limited samples, peer review, and so forth—the tools the *scientists* use to justify and establish trust in their findings. On this view, science education should be oriented to teaching scientific practices and argumentation. Only in this way, one may presume, can students become truly autonomous agents (e.g., Bergstrom & West, 2020; Daempfle 2013; Helfand, 2016; NGSS Lead States, 2013; Zimring, 2019).

How might this work in practice? Consider again the claim that 5G caused COVID-19. The astute, well-educated consumer would be aware of common flaws in reasoning. They would recognize that correlation does not necessarily indicate causation. One must control for possible confounders, such as population density on the maps. The map comparisons turn out to be coincidence, not evidence. So, too, for the case of vaccines. The correlation of vaccination with autism (ostensibly genuine) reflects the timing of the *diagnosis*, not a true physiological effect, as subsequent studies have shown. Plausible theories do not count as proven theories. Limited evidence does not demonstrate causal links and is not a basis for policy actions. Learning this level of reasoning seems simple enough, perhaps.

But consider the case of climate change. It is considerably more complex. One may surely introduce students to the Keeling curve (showing the steady rise in global carbon dioxide levels) and Mann’s “hockey stick” graph (showing the recent history of the planet’s temperature) and explain how they are important evidential benchmarks. But even these graphs are complex constructions that require quite a bit of technical reasoning—well beyond what the average citizen might master. In addition, the case for climate change spans numerous fields—paleoclimatology, marine biology, biogeography, atmospheric physics, thermodynamics, plant ecology, and so on. No one person can acquire enough skills in all those fields simultaneously. No one. Not even the scientists who contribute to that immense body of evidence. There is a boundary beyond which one cannot directly validate knowledge based on personal expertise. Not even professional scientists meet the educators’ ideal of the autonomous agent. They, too, depend on the specialized knowledge of others.

Science is not unique in this regard. Dependence on others for reliable knowledge arises where intellectual labor is distributed. No one can know everything. In our society of specialized expertise, we rely on doctors, lawyers, and dentists, as well as accountants, computer techs, meteorologists, electricians, plumbers, bridge welders, auto mechanics, appliance servicers, fire fighters, airplane pilots, military intelligence analysts, and so on. They each have specialized knowledge beyond our own (see also Nichols, 2017). As noted poignantly by philosopher John Hardwig (1985), we are *epistemically dependent* on each other.

Steve Norris (1995, 1997; Gaon & Norris 2000) cogently articulated the corollaries for science education (see also Griffiths, 1993). But over two decades later, educators have still generally failed to appreciate their significance. There is little evidence in curricula standards that the education system has fully grasped the problem of inevitable epistemic dependence or grappled with its consequences. Namely, the widespread goal of training autonomous thinkers is unattainable yet persists. The goal of full intellectual independence is, in principle, misplaced. Students need to learn instead the limits of personal knowledge. They need to develop “the wherewithal to deal intelligently with science and scientists despite their lack of scientific expertise” (1995, p. 202).

So, yes, the consumer may be able to dissect a simple argument and weigh simple evidence. But most scientific claims relevant to public policy or consumer choices are complicated. Like climate science, safety of waste incinerators or of fracking, cell phones and cancer, the environmental consequences of meat, weight-loss diets, and the efficacy of COVID-19 vaccines against variant strains. Without the relevant experimental know-how, the consumer cannot know if the evidence, *as reported to them*, exhibits technical competence and can be trusted. Without extensive experience in the field, they cannot know if the evidence, *as presented to them*, is sufficiently complete. Without background knowledge, they cannot know if there are alternative hypotheses and explanations and so be able to judge whether the reasoning is trustworthy. Thus, the consumer, as a non-expert, is inherently unable to fulfill the imagined role of an autonomous scientific agent. Indeed, this is why we train scientists through long study and apprenticeship—so that others can use their conclusions without having to do all the research and epistemic work themselves. The benefits of a cultural system of expert knowledge comes with a cost: epistemic dependence by non-experts.

In short, the consumer is not in a position to assess any scientific claim with the same epistemic assurance as the relevant scientific expert. The science consumer is, again, dependent on professional research scientists. Trying to assume the expert’s role by assuming that one has all the relevant evidence and background is not prudent. Indeed, that form of trust in oneself, ironically, opens the way to potential mischief (Hardwig, 1985, 1991). Imitators of science encourage the illusion that consumers can evaluate the evidence for themselves, while they feed them cherry-picked data and biased arguments (for examples, see Milloy’s *Junk Science Judo* and Murray, Schwartz and Lichter’s *It Ain’t Necessarily So*). They contrive an appearance of science from fragments of incomplete evidence and contorted plausibilities. It is an effort to short-circuit the communication from credible experts and thereby usurp the authority of authentic science. And therein lies much of the modern problem of a scientifically misinformed public: deception slips opportunistically into the gap of epistemic dependence.

The central lesson for the consumer, then, is to acknowledge our epistemic limits and indebtedness. The corresponding epistemic challenge is to assess the expertise of those who communicate the science. What matters is *not the evidence* but the *credibility* of whoever purports to speak for science. That is, to support functional public scientific literacy, educators need to focus foremost on the epistemics of science *in public discourse*. They cannot rely solely (or even mainly) on the epistemics of evidence and arguments that characterize “scientific practices” and the discourse among expert peers, wholly within scientific communities. Nor can they treat science news media as transparent or epistemically unproblematic. Students need to learn instead the distributed structure of knowledge and the social architecture of credibility (Allchin, 2012b; Goldman, 1999; Höttecke & Allchin, 2020).

Surprisingly, perhaps, science communication matters to the consumer *as much as the original science itself*. Indeed, from the perspective of the consumer who interfaces with science in the media (the consumer-centered view), the first goal must be (counterintuitively, perhaps) to disregard the evidence and, instead, establish the credibility of competing voices for science that crowd the media (see Fig. 1). Namely, the most immediate problem for someone situated amid the information maelstrom is *not* “what is the evidence?” but rather “who speaks for science?” *Who* are the credible experts and *who* will faithfully convey their consensus?

## Managing Mediated Expert Knowledge

As articulated by Hardwig (and echoed by Norris), virtually all knowledge is mediated (Sect. 3). But mediation opens the way to mischief. Bogus messages may enter the system. Misrepresentation and outright deceit are possible. An expert, applying their expertise, can likely notice any misstatements or errors. However, a non-expert has no such epistemic leverage. Someone who trusts science (at a general level) can easily be misled by someone purporting to be a spokesperson for science. Again, what is the citizen-consumer to do?

**Fig. 2:**
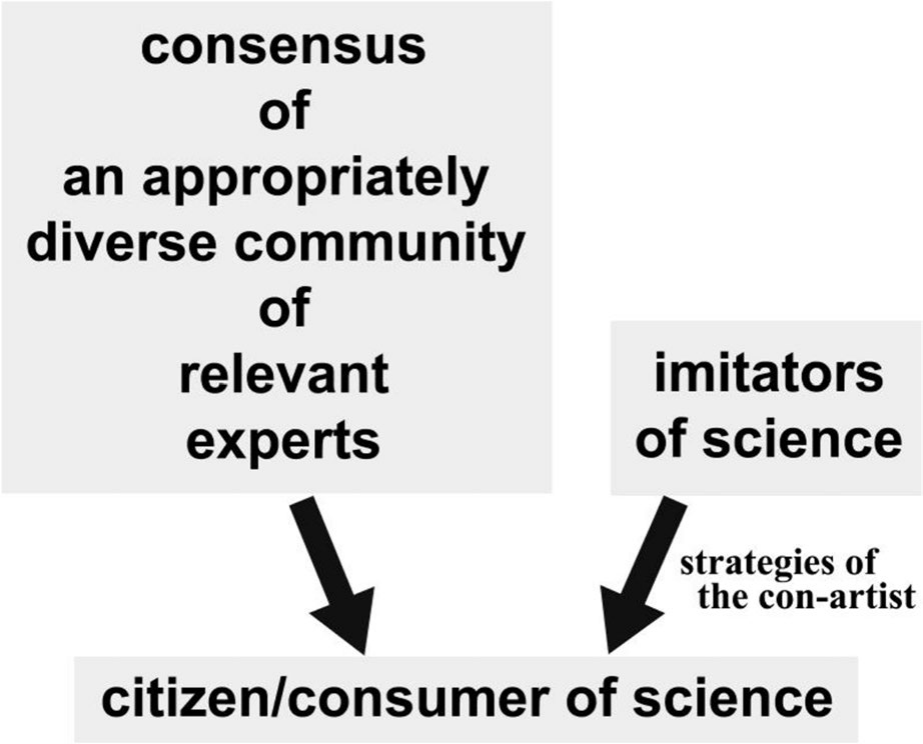
The challenge of deciding “who speaks for science?”

Here, the primary concern is no longer the details of how scientists develop their expert consensus. All the classroom lessons about the nature of science or scientific practices—using an exclusively internalist perspective—are of little use here. In this context, one may largely treat science as a “black box.” The concern in public discourse is downstream or post-consensus: how does the non-expert sorts the legitimate scientific consensus from the claims deliberately crafted to mimic science (Fig. 2)?

Educators might well turn to philosophers to help conceptualize the problem of epistemic trust. Not moral trust, contractual trust, or loyalty trust, but *epistemic* trust. For example, Gürol Izrik (2017), closely following Hardwig, recently presented the following characterization to a conference of science educators:

[Member of the public] M has warranted epistemic trust in [scientist] S as a provider of [proposition] P only if

1.
  S believes that P and honestly (i.e., truthfully, accurately, and wholly) communicates it to M either directly or indirectly.
2.
  M takes the fact that S believes and has communicated that P to be a (strong but defeasible) reason to believe that P.
3.
  P is the output of reliable scientific research carried out by S.
4.
  M relies on S because she has good reasons to believe that P is the output of such research and that S has communicated P honestly.
  (Izrik & Kurtulmus 2019, p. 1150)

This description certainly helps highlight how the core issue is honest communication—not understanding how science works. However, philosophical doublespeak hardly helps the science teacher. This long-winded (albeit perhaps impressive sounding) definition merely (re)states the problem. It conspicuously avoids articulating how to manage expert testimony in a concrete social context. Students need the practical details: (a) the nature of the relevant evidence for gauging honesty; (b) how to establish a speaker’s scientific expertise; (c) how to manage the collective nature of scientific research (the consensus, in contrast to a single expert’s opinion); (d) how one determines that someone will faithfully convey the scientific consensus; or (e) how to manage indirect communication (with multiple intermediaries, such as retweeting and reposting through social media). These are concrete social questions, not abstract philosophical ones.

**Fig. 3:**
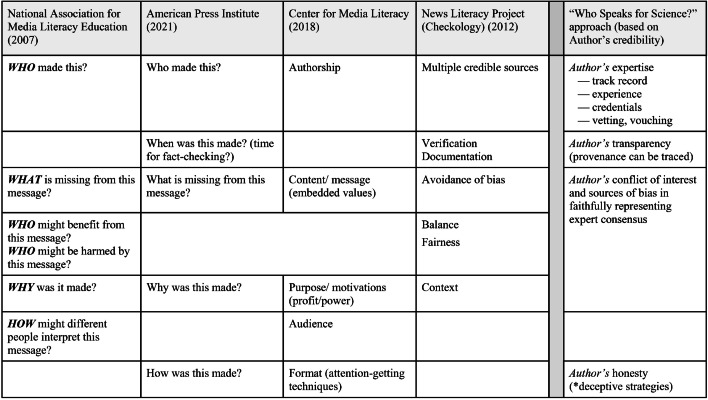
Comparison of ways to assess the trustworthiness of scientific claims in the news media. Conventional methods focus on documents and their epistemic attributes. The “who speaks for science?” approach focuses on the author and the sociology of their credibility

Given the inescapable role of communication, media literacy becomes central to interpreting trust in particular scientific claims (Höttecke & Allchin, 2020). One might thus tap the experience of journalists and other professionals in science news. In the past, the news media functioned as *gatekeepers*. That is, they selected relevant information, made it intelligible, and shaped it to be entertaining, all while trying to preserve its essential scientific content (Shoemaker et al., 2009; White, 1950). With the advent of the Internet and social media, however, the traditional role of gatekeepers has waned. Individuals are now increasingly confronting the challenge of filtering the reliability of sources of information on their own. In this changing environment, advocates of news media literacy have promoted several strategies for interpreting the credibility of claims, which might provide a helpful benchmark (see Fig. 3, first four columns; on principles used by science journalists, see National Academies of Science, Engineering and Medicine 2015, pp. 28–31). Unfortunately, most such checklists focus on features attributed to documents and written text. They sometimes refer to the need for credible sources but rarely articulate further the notion of credibility or its social context or how to manage efforts to mimic the conventional markers of credibility.

The question of the credibility of scientific claims in public discourse combines two major elements: expertise and honesty. Both refer *the person* who purports to speak for science. That is, the relevant evidence here is not about the scientific claim or the text but about the spokesperson. So, first, is the person qualified to vouch for the claim and its justification? Second, might they have reason to misrepresent the facts? These are the chief epistemic concerns for science in a public context (Fig. 3, last column). Notably, they focus on the author, not the claim in question. Moreover, they are not about the rhetorical structure, the imagery or emotional overtones, the medium, the technology, or social networks—all regarded as crucial by many other commentators.

As noted earlier, a critical feature of science not central to many other forms of public communication is expertise. Accordingly, assessment of credibility for scientific claims must be grounded foremost in the expert consensus. As articulated well by Oreskes (2019), several elements are relevant: (i) a credible public claim will reflect *consensus*; (ii) the consensus that matters is among *experts*; (iii) specifically, those with the *relevant expertise*; and, finally, (iv) the community should be *appropriately diverse* to allow an implicit system of checks and balances to balance individual biases (see also Harding, 1991; Solomon 2001; Ziman, 1968). (For an example of how each of these criteria was relevant during the recent pandemic, see Allchin (2020a). For how such concepts might be developed through student-centered classroom inquiry, see Allchin (2020b).)

In addition, one may further elaborate the notion of expertise. For example, one may consider several dimensions: the scientist’s track record, their experience, their credentials, and other forms of vetting or vouching by fellow experts. I leave these to be elaborated elsewhere (for an overview, see Allchin, 2012b, or Goldman, 2001).^1^

Usually, however, the honesty of the spokesperson is the first and most immediate concern. Claims, evidence, and arguments—even about the speaker’s own expertise—are meaningless if they are bogus from the start. For example, the citizen-consumer may discover conflicts of interest. The motives to mislead or deceive may be obvious. However, in many cases, speakers try to conceal any indicator that might alert the reader to doubt their credibility. Indeed, major efforts are often made to hide a lack of relevant expertise or dissent from a consensus or to project a misleading image of authority. Once again, the ultimate issue is honesty, addressed more fully in the next section.

## Science Con Artists

How can someone be successfully persuaded to believe bad science (Sect. 2)? The general trust in science opens a critical vulnerability. Many people will *say* that they speak for science. But that obviously does not mean they are either expert or honest. Some deliberately lie or mislead. They seek the authority of science without performing any of its epistemic work. They seek to bypass the task of persuading the expert scientific community and persuade the consumer-citizen directly. That is, such persons want to gain our trust, without having earned it. They hope to win our *confidence*. Hence, one may call them science “*con*” artists (Fig. 2).

Science con artists are not that different from shills and hucksters in other contexts. They use a familiar suite of stratagems. Most important, they exploit inherent cognitive dispositions and tendencies (Dobelli, 2013; O’Connor & Weatherall, 2019; Piatelli-Palmarini, 1994). Advocates of rationality often contend that those who succumb to these wiles are self-delusional. But, ironically, it is often the more intelligent persons—those who imagine that they are purely rational and therefore not vulnerable to such con artistry—who prove the most susceptible (Jackson & Jamieson 2007; Shermer, 2002, pp. 279–313). The unconscious habits are deeply embedded in our cognitive structure. Blaming people for being human is not very helpful. Yes, one may regulate these thought patterns. But it requires learning—and then being alert to the particular occasions when one needs to apply such learning. The con artist tries precisely to bypass our “BS alarms” and avoid triggering them. Their methods are designed to elicit *confidence*, establish psychological *ease*, and to foster an emotional aura of *trust*. Education seems the appropriate response.

Here, I describe five widely used tactics used to develop that *attitudinal* response as a context for belief (see Allchin, 2012a, 2015, 2018). Note that these focus on falsely establishing credibility and expertise rather than on any persuasive technique in the text, images, or videos.

First, there’s style. Science con artists need to *appear* trustworthy. They seem amiable and speak smoothly. That helps quell any intuitive sense of skepticism. The effect is primarily emotional. Feeling comfortable is, ironically, a warning sign of potential vulnerability. They likely wear nice clothes—or maybe a lab coat and eyeglasses, the stereotypical (yet also culturally quintessential) symbols of scientists. Websites look professional. Videos resemble public television documentaries. Books have high-quality publication standards. It is amazing how we can be beguiled by someone who merely looks the part. To avoid being influenced, a consumer-citizen needs to learn about the power of these unconscious psychological tendencies. They need to cross-check their own emotions (Rampton & Stauber, 2001, pp. 291–294; Freedman, 2010; Kahneman 2010).

A second tactic is outright disguise. To impress others as scientific, imitators don the markers of good science. For example, because we expect data, they use graphs and charts. They may use—then “graciously” explain—intimidating jargon. To achieve scientific credibility, one begins by enlisting a scientist to make the claim, even if that person does not have the *relevant* expertise. Can a nuclear scientist really speak authoritatively about second-hand smoke? And acid rain, too? *And* the ozone layer? *And* climate change? No (Oreskes & Conway, 2010). Alternatively, if publication in a peer-reviewed journal is the standard of credibility, then one hires ghostwriters from university faculty or medical schools (McGarity & Wagner, 2008, pp. 76–79; Rampton & Stauber, 2001, pp. 200–201). Much like plagiarism, only in reverse. Or one creates whole journals that one can portray as peer reviewed but that do not meet customary community standards (Michaels, 2008, pp. 53–55; Oreskes & Conway, 2010, pp. 244–245). If the voice of ordinary citizens is paramount, then corporate interests mimic “grass roots” organizations (Tabuchi, 2020). If scientific consensus is important, one enlists a dissenting scientist to provide an illusion of consensus, say, by giving congressional testimony (see the case of atmospheric scientist John Christy [2013] testifying about climate change). If one needs to appear to disavow bad science, one just calls the good science “junk” or disparages it as tainted by partisan politics (e.g., Steve Milloy’s *Junk Science Judo*; Murray, Schwartz & Lichter’s *It Ain’t Necessarily So*; or Berezow & Campbell’s *Science Left Behind*). Layers and layers of subterfuge. It’s all a disguise by bogus experts, who want others to believe that they are speaking on behalf of science.

A third strategy of science con artists in developing trust is exploiting social emotions. That is, our beliefs are shaped by those around us. We sometimes “calibrate” our views against those of our chosen peers. Our minds are motivated by social acceptance, as much as by any standard for reliable knowledge. Thus, psychologically, people tend to align their ideas and values to “fit in” and show allegiance to their group, whether based on ideology, identity, or politics (e.g., Kahan, 2013, 2017; Kahan et al., 2011; Kraft et al., 2015; O’Connor & Weatherall, 2019; Pennycook, McPhetres, et al. 2021). Con artists thus foster in-group sympathy and allegiance and out-group fear. For example, in 1999, the South African government denied that a virus caused AIDS. The public health minister Manto Tshabalala-Msimang drew on lingering anti-colonialist sentiments when she denounced antiretroviral drugs offered by developed nations as harmful. Local customs in nutrition, embedded in African culture and history, she claimed, would effectively combat the “alien” disease (Goldacre, 2010). Or a US political leader refers to SARS-Cov-2 as “the China virus,” thereby stoking xenophobic fears that eclipse the unjustified science (Hswen et al., 2021). The anti-fluoridationists in the USA (mentioned above) were united as much by shared anti-government sentiments, as by any science (Martin, 1991; Toumey, 1996). In the same way, anti-vaxxers and flat-Earthers form tight social groups that reinforce conformity and exclude dissenters. Likewise, communities where neighbors’ livelihoods depend on fossil fuel production tend to share deep skepticism about climate change (Harmon 2007; University of Kansas 2017). Social cohesion can be a powerful factor in belief. Accordingly, social media—by exploiting existing social networks—have helped amplify the reach of science con artists, aggravating the problem.

A fourth major stratagem is to manufacture doubt. Where one cannot win a scientific contest, one may still succeed at sowing discord or uncertainty. It is a powerful technique for scuttling any policy based on science, especially regulation of public or occupational health or management of environmental risks, as superbly documented by Michaels (2008, 2020) and Oreskes and Conway (2010). You might hear, “But we just don’t know for sure! We need *more research* before we can justify any action!” The appeal appears pro-science. But it deliberately evokes fears of being wrong. Doubt becomes uncertainty. Uncertainty becomes lack of proof, which then becomes error. By now, this has become a standard playbook for discounting established science (Kenner, 2015). Hence, when some highly predictable environmental “accident” occurs, you may hear industries opine, “We didn’t know! How was anyone to know?” (Consider the cases of the Fukushima nuclear plant disaster in Japan in 2011 or several dam failures in Uttarakhand, India, in 2013, in Laos in 2018, and in Brazil in 2015—and again in 2019. More misinformation from science con artists, here cloaked in the emotional guise of uncertainty and vulnerability.

A fifth and final tactic, when all else fails, flood the media. Create an illusion of the “wisdom of the crowd.” This elicits the tendency, guided by social emotions (noted above), to align with group norms. Lies spread easily on the Internet and social media and acquire apparent legitimacy merely through repetition and familiarity (Petersen et al., 2019; Vosoughi et al., 2018). Multiple websites host variants of the same disinformation (Tabuchi, 2020). Echoed by talk radio, Twitter, and YouTube (e.g., Avaaz, 2020). When special interests purchase or take control of newspaper chains, television networks, or media companies, they can easily forsake the news media’s traditional responsibility as stewards of public knowledge in order to promote private and ideological agendas (Alba & Nicas, 2020). All founts of propaganda. All bypass journalistic critique and the gatekeeping filters of expertise. Bogus science can reach far and wide, with con artists appearing to speak for science. The voice of authentic science, even if available, is left in a virtual *media shadow*. The growing popularity of the Internet and the ease of sharing via social media have only amplified the problem.

Where science enjoys cultural authority, those seeking power are motivated to imitate science. They may use any of these five tactics (or others) to help promote a substitute version of scientific information in their favor. Note that these particular stratagems are not just logical fallacies or lapses in sound reasoning, solved by standard critical thinking. A non-expert cannot expose the flaws by analyzing the argument or identifying some telltale feature of the text. Rather, they are fundamentally about honesty in communication. Like commercial advertising, the con artists appeal to emotions and attitudes that tend to engender sociality and disarm normal critical faculties. Dishonest, but persuasive nonetheless.

Con artists are less effective, however, when the intended target is aware of the tactics and how they work. “Immunity by inoculation” is the apt metaphor. Others call it preemptive debunking or “prebunking.” Studies have now shown that students and others can become more resistant to deceptive practices by learning about them in advance (e.g., Basol et al., 2021; Cook et al., 2017). Here, then (finally), is an apparent major component to solving the problem of bogus claims displacing consensus science: exposing the strategies that create an illusion of credibility, in part by focusing on “who speaks for science” rather than on what is said (the argument, the evidence, or the rhetoric). Thus, it would seem important to teach about the social tactics of con artists to students, with ample illustrations, as part of developing science media literacy.

## Summary

In this paper, I have provided evidence from contemporary and historical cases that many widespread educational views about the rejection of consensus science are misinformed. Adopting a consumer-centered perspective, one can see that the problem is less about trust in science than about deceptive communication practices and who is regarded as speaking for science (Sect. 2). While an understanding of and skills in scientific practices (or how science works, internally) are valuable, they are insufficient to address the challenge of untangling science communication in public discourse. A non-expert, however well trained, is epistemically ill-equipped to assess sophisticated and domain-specific evidence and argument and must instead rely on the consensus of the relevant experts (Sect. 3). Because acquiring such knowledge is mediated, the primary concern is interpreting the credibility of anyone who claims to be a voice for science. This involves socially measuring expertise and, most immediately, honesty (Sect. 4). Citizen-consumers thus need to learn about the many tactics used by would-be imitators of science to earn trust falsely—that is, to “innoculate” consumers to their adverse effects (Sect. 5). Accordingly, the guiding question for educators in addressing misinformation is not “why trust science?” but, more practically, “who speaks for science?” The remaining challenge, then, is to envision and assemble student-centered classroom inquiry activities that can help students encounter relevant cases and develop the concepts of epistemic dependence, credibility, expertise, gatekeeping, and so on, on their own in an active, constructivist mode (e.g., Allchin, 2020a, 2020b; Basol et al., 2021; Höttecke & Allchin, 2020; Proudfit, 2020; Zemplén, 2009).

## Data Availability

Not applicable.

## Footnotes

1 Scholarly work on the nature of expertise, both philosophical and sociological, has grown immensely in recent years. A good concise introduction is provided by Alvin Goldman (2001), who asks “Experts: which ones should you trust?” Other relevant work includes Goldman’s *Knowledge in a Social World* (1999), Selinger and Crease’s edited volume on *The Philosophy of Expertise* (2006), and Collins and Evans’ *Rethinking Expertise* (2007). In addition, Eyal (2013) offers an interesting analysis distinguishing the notions of experts (as a social role) and expertise (as an integrated network of resources and skills). Evans (2008) articulates a sociological concept of expertise that does not reduce to cultural relativism. Epstein (1995, 1996) shows powerfully how relevant experience can sometimes be more telling than formal credentials. For a more journalistic perspective, consult Freedman’s *Wrong: Why Experts Keep Failing Us* (2010) and Rampton and Stauber’s *Trust Us, We’re Experts!* (2001), especially on how—or when—experts can be wrong. All these sources can help provide the educator with the relevant conceptual background.
